# SODD Promotes Lung Cancer Tumorigenesis by Activating the PDK1/AKT and RAF/MEK/ERK Signaling

**DOI:** 10.3390/genes14040829

**Published:** 2023-03-30

**Authors:** Fan Bao, Su An, Yang Yang, Tian-Rui Xu

**Affiliations:** 1 Faculty of Environmental Science and Engineering, Kunming University of Science and Technology, 727 Jingming South Road, Kunming 650500, China; 2 Faculty of Life Science and Technology, Kunming University of Science and Technology, Kunming 650500, China

**Keywords:** SODD, gene knockout, PDK1/AKT, MAPK, lung cancer

## Abstract

Background: The Bcl2-associated athanogene4 (BAG4/SODD) protein could be identified as a tumor marker for several malignancies and plays a major role in the occurrence, development, and drug resistance of tumors. However, the role of Silencer of death domains (SODD) in lung carcinogenesis is still elusive. Objective: To illuminate the effect of SODD on the proliferation, migration, invasion, and apoptosis of lung cancer cells and tumor growth in vivo and explore the corresponding mechanism. Methods: The expression of SODD in tumor and normal tissues was determined and compared via western blot. *SODD* gene knockout lung cancer cells (H1299 cells) were established through a CRISPR/Cas9 gene deleting system, and a transient SODD overexpression of H1299 cells was also constructed. Then, cell proliferation and invasion were assessed through colony formation and cell counting kit-8 assays, transwell migration assays, and wound healing assays. Cell drug sensitivity is also analyzed by Cell Counting Kit-8 assay. The flow cytometer was used to perform cell circle and apoptosis analysis. The interaction of SODD and RAF-1 was confirmed by co-immunoprecipitation, and the phosphorylated level of Phosphatidylinositol 3-kinase (PI3K), Serine/threonine-protein kinase (AKT), Rapidly accelerated fibrosarcoma (RAF)-1,and extracellular signal regulated kinase (ERK) in cells was examined by western blot to evaluate the activation of PI3K/PDK1/AKT and RAF/MEK/ERK pathways. In vivo, Xenograft tumor assay of *SODD* knockout H1299 cells was used to evaluate further the role of *SODD* on the proliferation of H1299 cells. Results: SODD binds to RAF-1 and is over-expressed in lung tissues, and promotes the proliferation, migration, invasion, and drug sensitivity of H1299 cells. The reduced cells in the S phase and increased cells arrested in the G2/M phase were found in *SODD* knockout H1299 cells, and more cells got apoptosis. The expression of 3-phosphoinositide-dependent protein kinase 1(PDK1) protein in SODD knockout H1299 cells decreases distinctively, and the phosphorylated level of AKT, RAF-1, and ERK-1 kinase in *SODD* knockout H1299 cells is also less than that in normal H1299 cells. In contrast, SODD overexpression significantly increases the phosphorylation of AKT. In vivo, SODD promotes the tumorigenicity of H1299 cells in nude mice. Conclusions: SODD is overexpressed in lung tissues and plays a considerable role in the development and progression of lung cancer by regulating the PI3K/PDK1/AKT and RAF/MEK/ERK pathways.

## 1. Introduction

Lung cancer is one of the fastest growing malignancies with distinct invasiveness and migration globally. It ranks first in morbidity and mortality among malignancies [1]. Molecular targeted therapies for lung cancer can accurately attack tumor cells without harming normal cells, attracting increasing attention in the clinical therapy of lung cancer. Mitogen-activated protein kinase (MAPK) plays an important role in the development of lung cancer, among which k-ras and RAF-1 are studied in-depth in targeted therapy [2,3]. RAF-1 mutation is closely associated with small-cell lung cancer and head and neck malignancy. Therefore, the regulation of RAF-1 kinase has become one of the most important means to treat cancer [4]. Notably, RAF-1 depends not only on the activation of ERK but also on its associated proteins to play a role in tumorigenesis [5].

The Bcl-2-associated athanogene (BAG) protein’s family of six members was found in human cells, which are widely involved in the regulation of physiological and biochemical reactions. These included cell stress response, apoptosis, nerve tumor differentiation, cell cycles, and so on [6]. Among them, BAG1, BAG3, and BAG4 (also named SODD) may be associated with BCL-2 and correlated with the development processes of some cancers [7,8]. All the members share a conserved motif located in the C terminal, called the BAG domain (BD), which binds to the Hsp70/Hsc70 family proteins and modulates their activity [9,10]. BAG1 is competitively associated with RAF-1 or HSP70 via α1α2 or α2α3 helix of the BD, respectively, and then activates RAF-1 in a RAS-independent pathway. An activated RAF-1 activates its downstream target MEK and further promotes cell growth [11,12,13]. In addition, BAG3 can abrogate the proteasome degradation of RAF-1 mediated by HSP70 to maintain its level [14]. Previously, via co-immunoprecipitation and mass spectrometry (MS) identification, we found that SODD could interact with RAF-1 [15]. SODD has a shortened BD that is functionally similar to that of BAG1 [16]. We, therefore, speculated that SODD possibly binds to RAF-1 via the α2α3 helix to activate RAF-1 and its downstream pathways and positively regulates this pathway. Furthermore, if SODD can promote the phosphorylation of RAF-1, like BAG1, it will be likely to activate the RAF-1-mediated Bcl-2-related anti-apoptosis pathway. 

In this study, we examined the effect of SODD on H1299 lung cancer cell proliferation, migration, invasion, apoptosis, and tumor growth in vivo. Our results indicate that SODD promotes H1299 cell proliferation, migration, drug resistance, and invasion and that SODD knockout induces more cells to be arrested in the G2/M phase and apoptosis. Mechanically, SODD can be associated with RAF-1 to activate ERK and positively regulate the activation of the PDK1/AKT pathway.

## 2. Materials and Methods

### 2.1. Tissue Specimens and Ethics Statement

Human lung cancer tissue and matched normal adjacent tissue samples were obtained stochastically from 12 patients of the First People’s Hospital of Yunnan Province (Kunming, China) during 2015–2019. These samples were diagnosed as lung cancer tissue by the pathologist. The patients were well informed of the experimental processes and did not receive any chemotherapy, radiotherapy, or other adjuvant therapy. Written informed consent was obtained from all participants, and all protocols were approved by the Institutional Review Board of Kunming University of Science and Technology.

### 2.2. Cell Lines and Antibody Reagents

H1299 cell lines were purchased from the Cell Bank of China Academy of Sciences (Shanghai, China) and checked for mycoplasma contamination. Cell lines were cultured in RPMI-1640 Medium with 10% fetal bovine serum (Hyclone, Logan, UT, USA), containing 10 µg/mL streptomycin sulfate and 100 µg/mL penicillin G. Cells were cultivated in a chamber with 95% humidity and 5% CO_2_ at 37 °C.

Primary mouse anti-SODD antibody was purchased from Santa Cruz Biotechnology, Inc. (Santa Cruz, CA, USA), and mouse anti-GAPDH was purchased from Proteintech (Rosemont, IL, USA). Other antibodies, including rabbit anti-ERK, rabbit anti-p-ERK, and rabbit anti-p-RAF-1(S338), were purchased from Cell Signaling Technology Inc(Shanghai, China). Rabbit anti-PDK1, rabbit anti-p-AKT(T308), rabbit anti-N-cadherin, and rabbit anti-E-cadherin were purchased from Abcam (Waltham, MA, USA). Antimouse or antirabbit HRP-conjugated secondary antibodies (Santa Cruz Biotechnology Inc.) and MEK-specific inhibitor PD0325901 (Selleck Chemicals, Houston, TX, USA) were used.

### 2.3. Plasmid Construction and Cell Transfection Generation

To construct SODD knockout (SODD-KO) H1299 cells, a CRISPR/Cas9 gene deleting system consisting of a Cas9 enzyme cDNA and a gRNA (5′-GGGCGAAGAGGATATAAGGG-3′) in a pSpCas9 (BB)-2A-Puro (pX459) (Addgene, Berkley, MI, USA) was established to target the first exon of the *SODD*. The constructed plasmid was mixed with Lipofectamine 2000 (Invitrogen, Carlsbad, CA, USA), according to the manufacturer’s instructions, and transfected to H1299 cells. The transfected H1299-WT (H1299 wild type) cells were treated with (10 μg/mL) puromycin to screen stable SODD-knockout cells. The selected homozygous clones were further verified by DNA sequencing and western blot (WB).

To establish an SODD overexpressed plasmid, the total RNA of H1299 cells was extracted using MiniBest Universal RNA Extraction kit (TaKaRa, Dalian, China;) and was then reverse-transcribed into cDNA using PrimeScript™ RT-PCR kit (TaKaRa, Dalian, China) in accordance with instructions. Then SODD gene was amplified using cDNA as a template through the primers containing KpnI/NotI restriction endonuclease sites and an HA label (forward primer 5′-CGGGGTACCCGGACC ATGTACCCATACGACGT CCCAGACTACGCTTCGGCCCTGAGGCGCT-3′ and reverse primer 5′-TAAA GCGGCCGCTCATAATCCTTTTTTTTCTAAT-3′). The purified target fragment was digested with KpnI/NotI and inserted into the corresponding sites of a pcDNA5/FRT vector (Invitrogen) to generate a pcDNA5/FRT-HA-SODD plasmid. The pcDNA5/FRT-HA-SODD plasmid was transfected into WT H1299 cells, and the SODD expression levels in the transfected cells were examined by WB. All experiments were run in triplicate.

### 2.4. Co-Immunoprecipitation and WB Analysis

Total protein samples from the cultured cells were prepared using RIPA buffer (Pierce, Rockford, IL, USA) containing a protease inhibitor cocktail. Each of the protein samples was equal in quality and was separated by SDS-PAGE electrophoresis. They were then transferred to the PVDF membrane (Millipore, Burlington, MA, USA). The membrane was blocked in skimmed milk for 2 h and incubated with a primary antibody overnight at 4 °C. Subsequently, the membrane was washed twice with Tris-buffered saline with 0.05% Tween (TBST) and incubated with TBST containing the second antibody for two hours. The labeled protein bands were visualized and assessed via a Gray value analysis using ImageJ. Co-immunoprecipitation trials were performed according to the methods we used before [17].

### 2.5. Colony Formation Assays and Wound Healing Assays

The well-counted cells were inoculated into six-well plates (1000 cells/well) and cultured for two weeks. Cell growth was observed, and the medium was replaced every 2–3 d. As each clone contained about 50 cells, the medium was discarded. Cells were cleaned, fixed with 4% paraformaldehyde for 30 min, stained with crystal violet for 15 min, and washed with PBS two to three times to remove the background color. The stained clones were quantified and statistically analyzed.

For the wound healing assays, cells were grown into a monolayer formation and introduced to a serum-free medium for 12 h. A linear wound was made in the cell layer using a Pasteur pipette. The cells were washed thrice to remove detached cells and incubated in the medium containing 10% serum and mitomycin C (10 µg/mL) at 37 °C. Thereafter, the wound size was observed and measured at 0, 24, and 48 h.

### 2.6. Cell Invasion Assay

The cell invasion was evaluated through Transwell Migration Assays. Cells (3 × 10^5^ cells treated by mitomycin C [10 µg/mL] in 300 µL of serum-free medium) were incubated at 37 °C in the upper chamber. A mixture of 600 µL of DMEM containing 10% FBS and 10 µg/mL mitomycin C were added to the lower chamber. We removed the medium after 48 h and washed it twice with PBS. The cells in the lower chambers were fixed by formaldehyde (3.7% in PBS) at 27 °C for 2 min, stained with crystal violet, and photographed. The reversed cells in the upper chamber were removed.

### 2.7. Cell Cycle Analysis

The H1299 and H1299-SODD-KO cells were trypsinized, washed with PBS, and fixed in ice-cold 75% ethanol for 8 h. Thereafter, the samples were centrifuged and removed from the ethanol and exposed to 100 mg/mL RNaseA (Sigma-Aldrich, St. Louis, MO, USA) for 30 min. The cellular DNA was stained with propidium iodide (PI) (Sigma). A BD FACSCalibur flow cytometer was used to determine the cell−cycle distributions.

### 2.8. Cell Drug Sensitivity Post Anticancer Drug Treatment

H1299 cell drug sensitivity following Cisplatin treatment was determined using a Cell Counting Kit-8 (CCK-8) assay to evaluate how drug concentrations influenced cell growth. The logarithmic growth cells were seeded into a 96-well plate at 4 × 10^3^ cells/100 µL per well and incubated at 37 °C overnight. They were treated with increasing Cisplatin concentrations for 36 h. After the treatment, 10 µL of CCK-8 solution (Beyotime Biotechnology, Shanghai, China) (10 mg/mL) was added to each well, and the absorbance was measured at a double wavelength of 450 nm and 630 nm using a microplate reader (BMG LabtechPHERAstar FS, Offenburg, Germany)after 2 h of incubation. 

### 2.9. Cell Apoptosis 

H1299 cells were harvested and stained with Annexin V-fluorescein isothiocyanate (V-FITC) and PI and analyzed using the BD FACSCalibur system. The Cell Quest software v.5.1 was used to analyze the final data.

### 2.10. Cell Proliferation Assay

Cells were seeded into 96-well plates at a density of 2000 cells/100 μL per well and cultured in an incubator with a humidified 5% CO_2_ at 37 °C for 12 h, and 10 μL of CCK-8 solution was added. Subsequently, the 96-well plate was incubated in the incubator for one hour. A microplate reader was used to measure the optical density (Absorbance) per well at 450 nm and 630 nm, and this data was recorded as the data of “0” day. Cells were cultivated for 1, 2, 3, and 4 days later; the same way was used to measure the optical density per well. Then, the ratio of the optical density on each day to the average optical density at “0” day was calculated and analyzed statistically.

### 2.11. Animals

Twelve three-week-old male nude mice were fed standard sterilized food and water. Then 0.2 mL of WT and SODD knockout cells were subcutaneously injected into the legs of two groups of mice, respectively, at a density of 5 × 10^7^ cells/mL. Tumor formation was monitored using vernier calipers to measure tumor diameter (volume = width × length^2^/2) every 2 d. Fifteen days later, the mice were all sacrificed, and the tumors were removed for weighing. Tumor samples were fixed and embedded in paraffin, and hematoxylin and eosin (HE) staining was performed. Thereafter, the tumor samples were dewaxed, rehydrated, and blocked using 0.3% hydrogen peroxide in methanol for 30 min at room temperature. Samples were incubated at 4 °C overnight with an anti-PDK1 antibody (diluted 1:100). Slides were incubated with the rabbit secondary antibody and were counterstained with hematoxylin (The animal experiment is accomplished by Kunming Parui Biological Technology Co. Ltd., Kunming, China).

### 2.12. Statistical Analysis

Lung cancer and the adjacent normal tissue groups were statistically compared using an unpaired *t*-test. The multiple *t*-test was applied to examine whether there were significant differences in the number of cells and colonies. The WB band density was measured using the Image-Pro Plusv.6.2 software(Media Cybernetics, Rockville, MD, USA). Data was analyzed using the GraphPad Prism 8.0.2 software(GraphPad Software Inc., San Diego, CA, USA). Statistical differences were considered significant at *p <* 0.05.

## 3. Results

### 3.1. SODD Binds to RAF-1 and Is Over-Expressed in Lung Tissues

**Figure 1:**
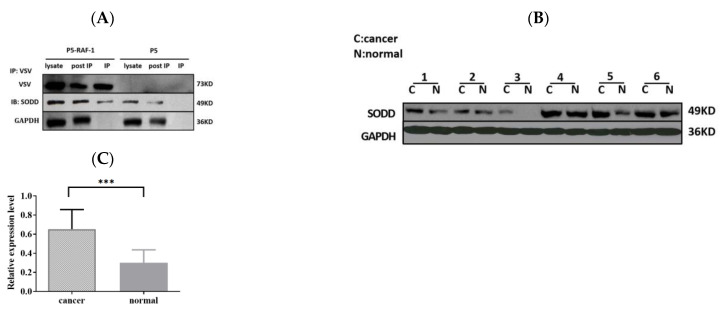
SODD is confirmed as an RAF-1-associated protein and overexpressed in lung cancer tissues. (**A**) VSV-tagged RAF-1 was inserted into the pcDNA5 plasmid (P5) in HEK 293T cells, and VSV beads were used to catch RAF-1-binding proteins. The WB outcome confirms that SODD binds to RAF-1. (**B**,**C**) SODD expression in lung cancer tissues was analyzed by WB. The expression of GAPDH was detected to ensure the samples’ loading amount. The SODD expression significantly increased in cancer tissues, as compared with adjacent normal tissues. (*** *p <* 0.001).

Previously, we identified 272 specific binding proteins of RAF-1; SODD was one related to the tumor process [15]. We confirm the interaction of SODD and RAF-1 by using the co-immunoprecipitation in this study (Figure 1A).

We compared SODD expression in human tissue samples that were collected from the First People’s Hospital of Yunnan Province of China and found that SODD expression was upregulated in lung cancer tissue (Figure 1B,C).

### 3.2. SODD Promotes the Proliferation, Migration, and Invasion of H1299 Cells In Vitro

**Figure 2:**
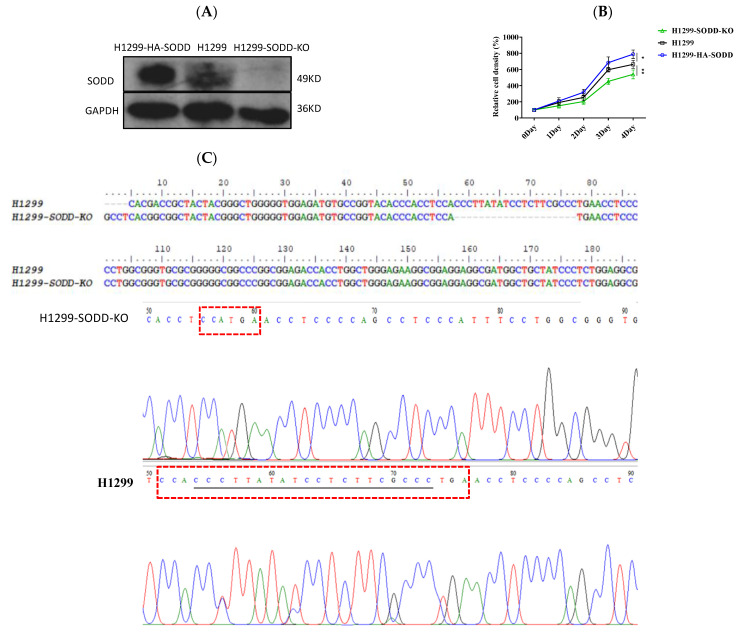
Establishing a SODD knockout (KO) and overexpressed (HA-SODD) H1299 cell line and exploring the effect of SODD on H1299 cell growth. (**A**) SODD expression in SODD (KO), HA-SODD, and H1299 cells was detected by Western blotting (**B**) SODD promotes the proliferation of H1299 cells. The overexpression of SODD could promote the growth of H1299 cells, whereas the knockout of H1299 could inhibit it. (* *p* < 0.05, ** *p* < 0.01 vs. control) (**C**) The DNA sequencing of SODD-KO (SODD−/−) and WT H1299 cells served to confirm SODD knockout. The missing bases are underlined in the red box.

**Figure 3:**
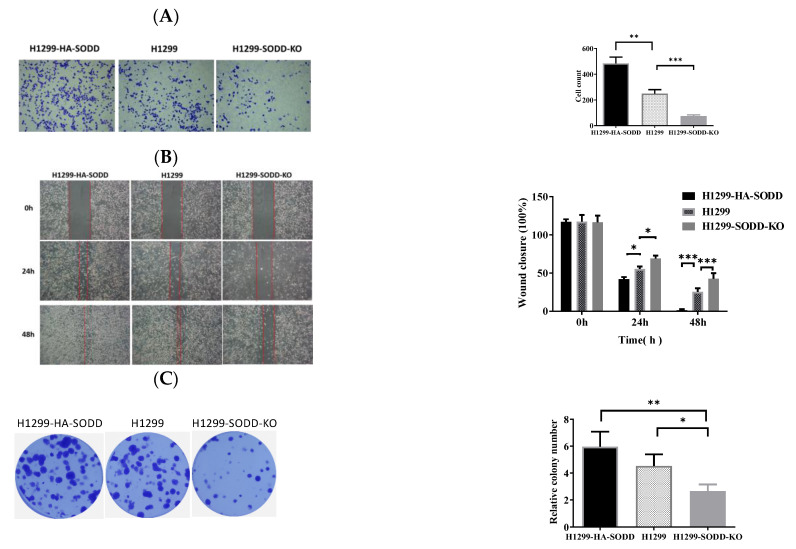
SODD promotes the migration and invasion of H1299 cells. (**A**) Transwell assay: HA-SODD H1299, SODD knockout H1299, and H1299 cells migrating through the Transwell chamber after 48 h were counted. (**B**) Wound healing assay. The represented images of the relatively migrated speeds of HA-SODD H1299, SODD knockout H1299, and H1299 cells at 0 h, 24 h, and 48 h, respectively. (**C**) The typical image of colony formation in HA-SODD H1299, SODD knockout H1299, and H1299 cells (* *p* < 0.05, ** *p* < 0.01, *** *p* < 0.001 vs. control).

**Figure 4:**
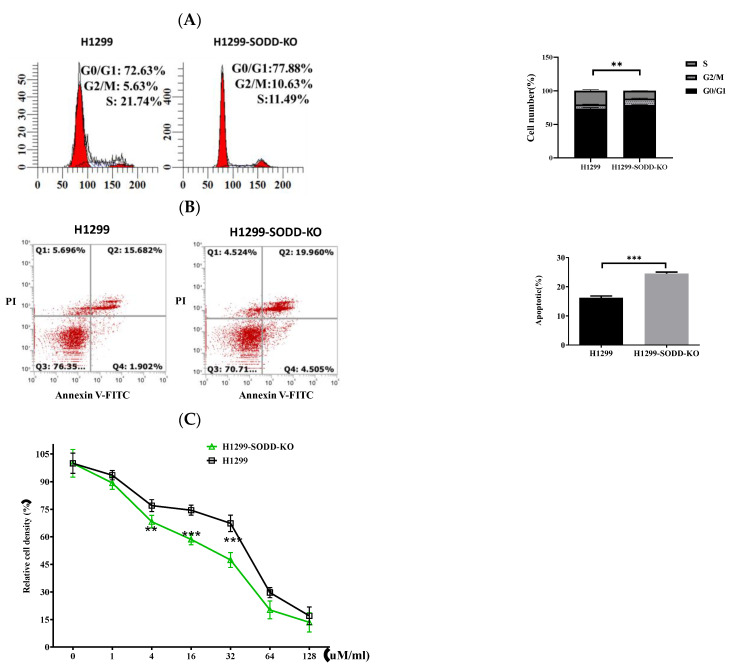
SODD knockout promotes apoptosis and cell cycle arrest in H1299 cells, reduces its ability, and enhances the sensitivity of H1299 cells to cisplatin. (**A**) The distribution of cells in G1/G0, G2/M, and S phases was measured through flow cytometry. (**B**) The percentage of apoptotic SODD-KO H1299 and H1299 cells. (**C**) SODD-KO H1299 and H1299 cells were incubated with increasing concentrations of cisplatin and counted through CCK-8. (** *p* < 0.01, *** *p* < 0.001 vs. control).

To determine the effects of SODD on lung cancer cell development, we first established a SODD deficient (SODD-KO) H1299 cell line by CRISPR/Cas9 gene editing and constructed a transient SODD overexpression (HA-SODD)H1299 cells. The knockout was confirmed by DNA sequencing and WB (Figure 2A,C). We subsequently quantified the proliferation in SODD (KO), HA-SODD, and WT H1299 cells. SODD deficiency significantly decreased the growth of H1299 cells, whereas the overexpression of SODD increased its growth (Figure 2B). Furthermore, HA-SODDH1299 cells form more clones than WT cells, indicating a stronger clone formation ability. Conversely, SODD-KO cells present fewer clone numbers (Figure 3C). The flow cytometry analysis showed that the proportion of WT cells in the S phase was nearly 10% higher than that of the SODD-KO H1299 cells and that SODD knockout induces more cells arrested in the G2/M phase (Figure 4A). These findings indicated that SODD knockout could inhibit H1299 cell proliferation.

The inhibition of SODD knockout on H1299 cell growth encouraged us to explore its further effect on lung cancer development. First, we performed a Transwell migration to evaluate the migration of H1299-SODD-KO cells. We found that SODD-KO H1299 cells that transferred to the lower chamber were significantly fewer than normal H1299 and HA-SODD cells. There were also more HA-SODD H1299 cells than WT cells that invaded the lower chamber of the Transwell (Figure 3A). Subsequent wound healing assays (Figure 3B) also consistently proved that SODD knockout would diminish the migrated ability of H1299 cells. Above all, these results strongly indicate that SODD plays a positive role in the invasion of H1299 cell lines.

### 3.3. SODD Inhibits Apoptosis of H1299 Cells and Enhances Its Resistance to Cisplatin

To explore the effect of SODD on the drug sensitivity of lung cancer, we also studied the susceptibility of H1299 cells to the anticancer drug cisplatin. SODD deficiency increases the sensitivity of H1299 cells to cisplatin (Figure 4C). This finding indicates that SODD could enhance the drug sensitivity of H1299 cells to pro-apoptotic agents. Flow cytometric analysis indicated that SODD deficiency increased the rate of apoptosis of the H1299 cells (Figure 4B). In addition, we demonstrated that nearly 10% less of the cells entered the S phase (Figure 4A) when SODD was knocked out. This indicated that SODD knockout could inhibit the proliferation of H1299 cells.

### 3.4. SODD Promotes the Activation of PDK1/AKT and MAPK Pathways

**Figure 5:**
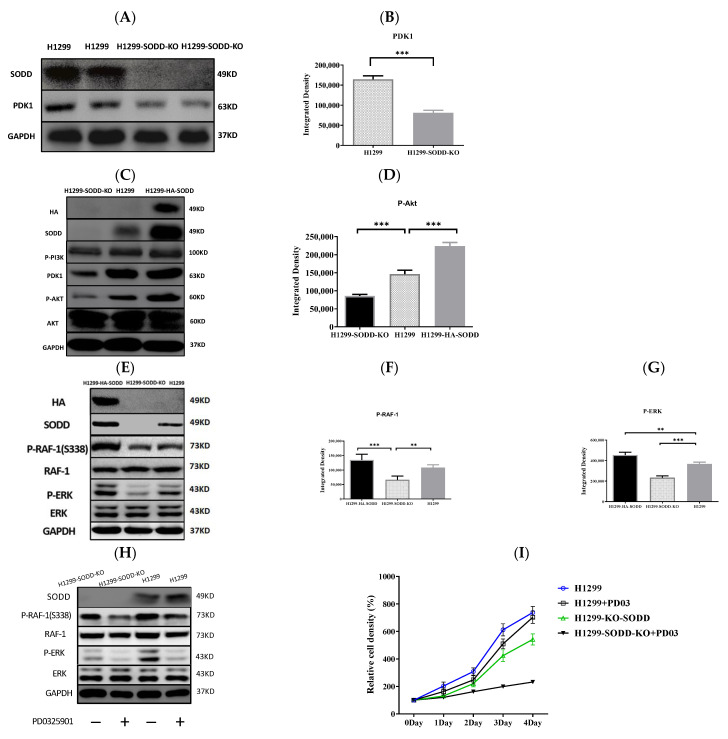
Effect of SODD expression on PDK1/AKT and RAF-1/ERK cascades. (**A**,**B**) PDK1 expression in the SODD-KO and WT H1299 cells was detected by WB. (**C**) WB shows the effect of SODD knockout on the PDK1/AKT signals in response to EGF. (**D**) Phospho-AKT (S308) in H1299-HA-SODD, SODD-KO, and WT H1299 cells. (**E**) WB shows the phosphorylation level of RAF-1 and ERK in SODD-KO, HA-SODD, and WT H1299 cells. (**F**,**G**) Phospho-RAF-1 and ERK in H1299-HA-SODD,SODD-KO, and WT H1299 cells. (**H**,**I**) WB shows the total and phosphorylated RAF and ERK in SODD-KO and WT H1299 cells treated with PD0325901 and the correspondent cell growth curve (H) (** *p* < 0.01,*** *p <* 0.001 versus control).

The RNA sequencing analysis on SODD-KO H1299 cells demonstrated that the transcriptional level of PDK1 in H1299-SODD-KO cells was remarkably less than that in normal H1299 cells. Accordingly, the expression of PDK1 protein in SODD-KO H1299 cells decreases distinctively (Figure 5A,B), and the phosphorylated level of AKT kinase in H1299-SODD-KO cells is also less than that in normal H1299 cells. In contrast, SODD overexpression significantly increases the phosphorylation of AKT (Figure 5C,D).

RAF-1 S338 phosphorylation was reduced in SODD-KO H1299 cells. Simultaneously, the ERK (downstream kinase of RAF-1 in the MAPK pathway) phosphorylation significantly decreased (Figure 5E–G). These findings show that SODD knockout inhibits the activation of RAF/MEK/ERK cascades. Subsequently, we used the MEK-specific inhibitor PD0325901 to further confirm the activation of MAPK by SODD.PD0325901 is a selective and noncompetitive MEK inhibitor that effectively inhibits ERK1/2 phosphorylation. After H1299 and SODD-KO H1299 cells were treated withPD0325901 for 24 h, the ERK phosphorylation in both cells was significantly reduced, and SODD-KO H1299 cells had the lowest level of ERK phosphorylation (Figure 5H). Accordingly, the proliferation of H1299 and SODD-KO H1299 cells treated by PD0325901 was also inhibited, and SODD-KO H1299 cells proliferated at the lowest speed (Figure 5I).

### 3.5. SODD Promotes the Tumorigenicity of H1299 Cells in Animal Trials

**Figure 6:**
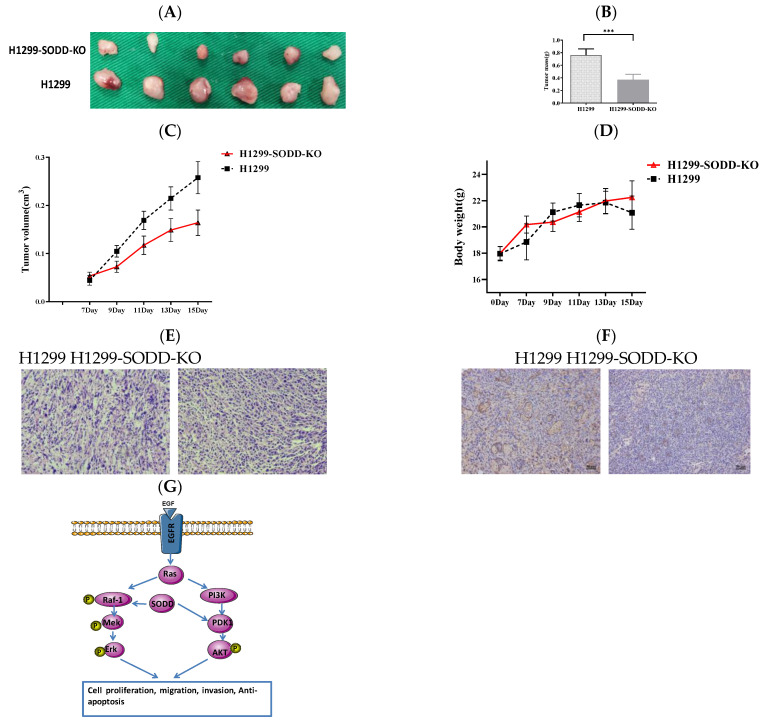
SODD knockout inhibits H1299 cell tumorigenicity in a nude mouse xenograft model (**A**) Two groups of excised tumors from nude mice injected with SODD-KO H1299 and H1299 cells. (**B**) The statistical difference in the tumor masses from the two groups (*** *p <* 0.001). (**C**,**D**) The difference in the tumor volume and body weight between two groups of mice (**E**) Hematoxylin and eosin (HE) staining of tumors from the two groups. (**F**) PDK1 staining of tumors from the two groups. (**G**) The indicated roles of SODD in the RAF-1/MEK/Erk and PI3K/PDK1/AKT pathways.

To further explore the effect of SODD on the tumorigenesis of lung cancer, we established a subcutaneous xenotransplanted tumor model of lung cancer. Legs of nude mice were injected with SODD-KO and matched WT H1299 cells. The process of tumor formation was then monitored. Fifteen days later, the tumor mass and volume of nude mice groups injected with SODD-KO H1299 cells were remarkably lower than that of the WT group (Figure 6A–C). However, there was no distinct difference in the body weight of the two groups of mice (Figure 6D). H&E staining of the xenograft tumor tissues suggested diminished tumorigenicity of SODD-KO H1299 cells in vivo (Figure 6E). Immunohistochemistry staining of PDK1 showed that the PDK1 expression in the SODD-KO xenograft tumor was remarkably lower than that of the WT ones (Figure 6F). When combined, these findings suggest that SODD can promote tumorigenesis of H1299 cells in vivo.

## 4. Discussion

SODD protein plays a “molecular switch” role in the TNF signaling pathway, which is a pronounced signaling pathway related to cell apoptosis. By modulating diverse signaling pathways, SODD appears to regulate the susceptibility of cell responses to drug stimuli. It is gradually utilized as a tumor marker and potential chemotherapy target in several tumors, including breast, pancreatic, and ovarian cancer [18,19,20,21]. In this study, SODD expression increased dramatically in lung cancer compared to the expression levels in normal lung tissue. This is consistent with the data from the UALCAN (http://ualcan.path.uab.edu/index.html (accessed on 18 January 2023)) database, which shows that SODD expression is significantly higher in lung squamous cell carcinoma and adenocarcinoma tissues than in normal tissue. To elucidate the effect of SODD on lung cancer cells, we constructed SODD knockout H1299 cell lines. SODD deletion significantly inhibited the proliferation (Figure 2B), invasion, migration (Figure 3), and cell circle progression (Figure 4A) of H1299 cells, as well as tumor growth in nude mice (Figure 6).

Although SODD is implicated in the progression of several cancers, the specific mechanism for it is still illicit. The PI3K/PDK1/AKT intracellular signal pathway involves mainly cell growth and anti-apoptosis by affecting the activity of downstream effect molecules and is closely related to the development and progression of human tumors [22]. SODD can bind to SKIP to inhibit the dephosphorylation of PI(3,4,5)P3 to PI(3,4)P2 and enhance AKT phosphorylation to promote the migration of COS-7 cells [23].Furthermore, overexpression of SODD in colorectal cancer cells increases AKT phosphorylation and enhances glucose uptake [24]. Our results show that SODD knockout leads to a significantly declined expression of PDK1 and reduced AKT phosphorylation. SODD overexpression conversely increases AKT phosphorylation in H1299 cells. PDK1 is an upstream activator of AKT kinase, and the phosphorylated site Thr308 of AKT is mainly phosphorylated by PDK1 [25]. During the last few decades, the inhibitions of the PDK1-AKT cascade have shown great potential as a target for cancer therapy [26,27]. Some putative miRNAs could regulate the progression of lung cancer through the PDK1/PI3K/AKT pathway [28,29]. Accumulating evidence indicates that the inhibition of PDK1—and not AKT—is more effective in the suppression of the PDK1/AKT/mTORC1 axis in some rare malignancies, and PDK1 is speculated to activate mTORC1 by pathways other than PDK1/AKT/mTORC. Indeed, the carcinogenicity of PDK1 is suggested to be mediated by other AGC family members except for AKT, including MEK, PKCα, and SGK3 [30,31,32]. Thus, the inhibition of PDK1 is more promising than AKT as a new target for cancer therapy. Our study demonstrates that the deletion of SODD could downregulate PDK1 expression in vitro and in vivo (Figure 6F) and decrease AKT phosphorylation. This indicates that SODD may be a target for the precise treatment of lung cancer.

We also show that SODD/SODD is associated with RAF-1 and promotes activation of the RAF/MEK/ERK cascade. SODD knockout in H1299 cells reduced the phosphorylation of RAF-1 and eventually inhibited the activation of the RAF/MEK/ERK pathway. Furthermore, the phenotypic changes in SODD-KO H1299 and H1299 treated by PD0325901 cells are similar. Mechanically, BAG1, a widely studied member of the BAGprotein family, is suggested to bind to the catalytic domain of RAF-1, possibly through its α-helix regions, to activate RAF-1 in a Ras-independent way [11]. Thus, we speculate SODD also could bind to the catalytic domain of RAF-1 through itsα-helix to activate RAF-1.

Cancer progression is associated with reduced apoptosis and increased proliferation of cells. SODD has a property that can antagonize apoptosis through the blocking or activating of several apoptosis-related signal pathways. SODD in bone marrow cells of acute lymphoblastic leukemia (ALL) children appears to be abnormally high, especially in the case of clinical drug resistance. Correspondingly, the downregulated SODD expression in leukemic cells increases the sensitivity of leukemic cells to drugs [33]. Eichholtz Wirth [34] found that SODD possibly plays a part in cisplatin resistance mediated by NF-κB. In our study, SODD increased the cisplatin resistance of H1299 cells, possibly by promoting anti-apoptosis. These findings are consistent with those of previous studies. Apart from blocking TNFR1-mediated apoptosis signaling, SODD may activate typical PI3K/AKT anti-apoptosis signaling, which may play a role in the anti-apoptosis of SODD. Future studies should focus on the influence of SODD on AKT downstream signaling molecules related to anti-apoptosis.

## 5. Conclusions

In a word, SODD may promote proliferation, invasion, metastasis, and drug resistance of lung cancer cells, where PI3K/PDK1/AKT and RAF/MEK/ERK signaling play a considerable role. SODD may be a new tumor indicator of lung cancer and could be a potential molecular target for lung cancer therapy.

## Data Availability

All data in this manuscript are available from the corresponding author upon reasonable request.
